# The effect of therapeutic listening on anxiety and fear among surgical patients: randomized controlled trial^1^

**DOI:** 10.1590/1518-8345.2438.3027

**Published:** 2018-08-09

**Authors:** Ana Cláudia Mesquita Garcia, Talita Prado Simão-Miranda, Ana Maria Pimenta Carvalho, Paula Condé Lamparelli Elias, Maria da Graça Pereira, Emilia Campos de Carvalho

**Affiliations:** 2 PhD, Adjunct Professor, Escola de Enfermagem, Universidade Federal de Alfenas, Alfenas, MG, Brazil.; 3 Doctoral student, Escola de Enfermagem de Ribeirão Preto, Universidade de São Paulo, PAHO/WHO Collaborating Centre for Nursing Research Development, Ribeirão Preto, SP, Brazil. Scholarship holder at Conselho Nacional de Desenvolvimento Científico e Tecnológico (CNPq), Brazil.; 4 PhD, Senior Professor, Escola de Enfermagem de Ribeirão Preto, Universidade de São Paulo, PAHO/WHO Collaborating Centre for Nursing Research Development, Ribeirão Preto, SP, Brazil.; 5 PhD, Physician, Hospital das Clínicas, Faculdade de Medicina de Ribeirão Preto, Universidade de São Paulo, Ribeirão Preto, SP, Brazil.; 6 PhD, Associate Professor, Escola de Psicologia, Universidade do Minho, Braga, Portugal.

**Keywords:** Interpersonal Relations, Nursing Care, Anxiety, Fear, Preoperative Care, Colorectal Neoplasms

## Abstract

**Objective:**

To investigate the effect of therapeutic listening on state anxiety and surgical fears in preoperative colorectal cancer patients.

**Method:**

A randomized controlled trial with 50 patients randomly allocated in the intervention group (therapeutic listening) (n = 25) or in the control group (n = 25). The study evaluated the changes in the variables state anxiety, surgical fears and physiological variables (salivary alpha-amylase, salivary cortisol, heart rate, respiratory rate and blood pressure).

**Results:**

In the comparison of the variables in the control and intervention groups in pre- and post-intervention, differences between the two periods for the variables cortisol (p=0.043), heart rate (p=0.034) and surgical fears (p=0.030) were found in the control group, which presented reduction in the values ​​of these variables.

**Conclusion:**

There was no reduction in the levels of the variables state anxiety and surgical fears resulting from the therapeutic listening intervention, either through the physiological or psychological indicators. However, the contact with the researcher during data collection, without stimulus to reflect on the situation, may have generated the results of the control group. Clinical Trial Registration: NCT02455128.

## Introduction

Global cancer deaths increased 57% between 1990 and 2013^1^. Colorectal cancer can be highlighted as one of the main causes of cancer-related deaths, and most cases of this type of cancer are treated surgically^2^. Hospitalization for surgery can generate anxiety and fear in patients, after all, the surgical procedures and the hospitalization represent a threat to the patients and their families due to physical changes, and psychological and social reactions to this situation^3^ ^-^ ^4^. The situation is aggravated when it comes to patients who will undergo oncologic surgery, since cancer is a cause of clinically significant suffering^5^.

Preoperative anxiety is a prevalent concern with deleterious effects on patient recovery, which can have repercussions such as increase in the use of anesthetic agents, heightened postoperative pain, and prolonged hospitalization^6^. According to the literature, the presence of preoperative fear was associated with increased pain rates, poor global recovery, lower levels of quality of life and vitality after surgery^7^ ^-^ ^8^.

Ability for managing negative emotional responses and distressing symptoms such as anxiety and fear is essential for the quality of life of a cancer patient. There are indications that psychotherapeutic interventions can contribute to the reduction of emotional distress and to the improvement of the quality of life of people with cancer^9^. Considering anxiety and fear as symptoms, rather than as disorders that would require treatment with longer lasting effects, they can be managed through brief interventions^10^. This can be achieved by different actions that are proven to be effective for reducing psychological comorbidities, such as therapeutic listening, also known as active listening^11^, which can be performed by nurses^12^.

Therapeutic listening is a communication resource that can be valuable in the care relationship^13^. It is characterized by the set of interactions occurred in the professional-patient relationship when the patient has the chance to talk about his apprehensions freely. It is a process in which the professional aims to help the patients to alleviate their anxiety and increase their adaptive capacity^14^. Aimed at assessing the use of listening as a support for therapeutic communication, this study was based on the Person-Centered Approach^15^. 

Despite of the recognized therapeutic value of listening^16^, studies on this subject are still scarce^17^. This motivated the search for its effect in a specific situation, the preoperative period of the therapeutic process of colorectal cancer. The importance of this study is related to the need to increase knowledge and broaden discussions on the use of therapeutic listening as a way to reduce anxiety and surgical fear, which are present in patients who are expecting a surgical procedure. The objective of this study was to investigate the effect of therapeutic listening on state anxiety and surgical fears in preoperative patients of colorectal cancer surgery. To do so, the variables compared in the pre and post intervention stages and in the control group (CG) and intervention group (IG) were physiological variables (salivary cortisol and amylase, heart and respiratory rates and blood pressure) and psychological variables (state anxiety scores and surgical fears) associated with feelings of anxiety and fear.

## Method

This is a prospective, parallel, open-label randomized controlled trial with equal allocation rate (1:1). 

The study participants were patients admitted for surgical treatment of colorectal cancer in the surgical clinic of a teaching hospital located in a city in the state of São Paulo (Brazil). For the calculation of the sample size, the State-Trait Anxiety Inventory (STAI) was used. Considering a difference of 10 points (δ) in the State-Trait Anxiety Inventory - Sate (STAI-S) score, a significance level of 5% (z1-α = 1,96) and a power of 80% (z1-β = 1.96), the result was 25 individuals for each group. The data related to the group variances was obtained by a procedure test, with correlation of 0.5.

Participants were eligible for inclusion if they: (a) were 18 years old or older, (b) were hospitalized for colorectal cancer surgery, (c) were not undergoing any other cancer treatment, (d) were not participating in another research (e) had a level of education that allowed reading and interpreting the instruments used in the study, which were self-reporting (f) were clinically well and/or stable (obtained score less than or equal to 3 in the Eastern Cooperative Oncology Group), and (g) presented a state anxiety score equal to or greater than 25 in the STAI-S in the first approach, which was performed previously and independently from the pre-intervention data collection. The cut-off point of 25 in the STAI-S was based on the findings of a study on the effects of complementary therapies on clinical outcomes in patients being treated with radiation therapy for prostate cancer^18^.

The exclusion criteria were: (a) having psychiatric disorders (identified in the patient’s medical record), (b) presenting metastasis and (c) using medication containing corticosteroids.

The discontinuity criterion adopted for this study was related to patients who were receiving procedures necessary for the surgery during the data collection process, such as the preparation of the intestinal tract. These patients were discontinued from the study.

In the IG, the patients were informed that they would have 30 minutes to talk to the researcher about their experience with hospitalization for cancer treatment (concerns, fears, doubts, or any other issue the patient wanted to treat). The interaction was initiated with the following guiding question: “How has your experience been in the hospitalization for the treatment of your disease?” Before the end of the therapeutic listening intervention, the patient was asked the following question: “Is there anything else you would like to talk about?” In the CG, patients were told they would have some data collected. Subsequently, the researcher would be absent for 30 minutes and, after this interval, would return for the conclusion of the research.

The data were collected from August 2014 to October 2015. Data collection schedules were previously set according to the hospital routine. Participants were admitted to a preoperative unit the morning of the day before surgery. Data collection occurred on the day of admission of the patients, who were invited to participate in the study after being informed about the purpose of the research. 

The data collection occurred in three moments: first approach, pre-intervention moment and post-intervention moment. In the first approach, carried out at 8a.m., when patients had already been assessed for eligibility according to the inclusion and exclusion criteria, the STAI and a questionnaire to characterize the participants were applied. After two and a half hours, in the second moment of the study (pre-intervention) the following data were collected: saliva samples for analysis of salivary cortisol and salivary alpha-amylase (SAA), heart rate (HR) and respiratory rate (RR), systolic blood pressure (SBP) and diastolic blood pressure (DBP), state anxiety, and surgical fears. One hour after the pre-intervention stage and shortly after the IG intervention and the CG, in the third and last moment of the study (post-intervention), the same variables were collected. In order to verify the presence of circadian rhythm of cortisol in the participants, two samples of saliva were collected, one at 8p.m. and the other at 11p.m., on the day before the surgery. The objective of the verification of the circadian rhythm was to identify if the participants of this study, patients with cancer, would present differences in this rhythm.

Participants were randomized into two groups: control and intervention. For this, a person who was not part of the activities developed in this research generated a randomized list in Excel 2007, which contemplated the CG and the IG. The sheets containing the descriptions “Intervention Group” and “Control Group” were each placed in opaque envelopes, sealed and opened by the main researcher after the data collection in the pre-intervention moment, when it was decided on which group the patient would be allocated. The instruments used in this study were answered by the patients themselves and the data referring to the physiological variables were collected by the researcher.

**Figure 1:**
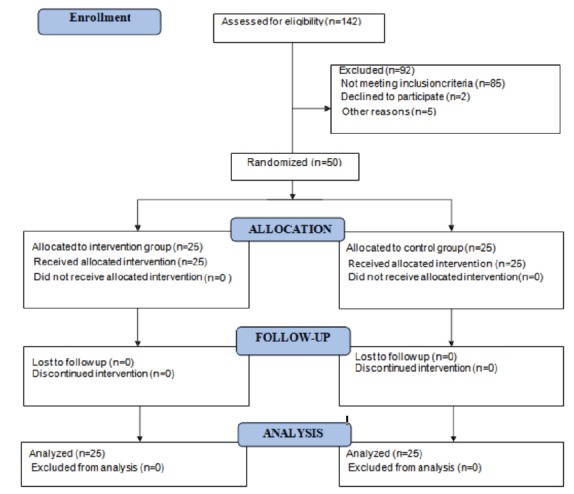
CONSORT 2010 Flow Diagram

During the data collection period, two participants declined to participate and five were discontinued due to routine hospital care activities during data collection. Thus, 50 participants reached the end of the study (Figure 1).

The following instruments were used for data collection:

Socio-demographic questionnaire: the socio-demographic variables collected were age, gender, level of education, marital status, monthly family income and religion;

State-Trait Anxiety Inventory: the anxiety was evaluated through the STAI^19^, validated in Brazil^20^. This instrument presents 40 items, of which 20 assess trait anxiety and 20 assess state anxiety in different constructs. To respond to the questionnaire the individual uses a Likert scale of four items ranging from 1 (not at all) to 4 (very much so). The score of each part varies from 20 to 80 points, and the scores may indicate low anxiety (20-30), moderate anxiety (31-49) and severe anxiety (greater than or equal to 50). Regarding the reliability of the STAI in this study, in the first approach, the results were α = 0.89 for the STAI-S and α = 0.83 for the State-Trait Anxiety Inventory - Trait (STAI-T), values ​​considered acceptable for this research^20^;

Surgical Fear Questionnaire (SFQ): Fears related to surgery were measured using the SFQ, which was validated by the researchers for use in this study, with the author’s authorization. The scale assesses surgical fears in 8 descriptive statements divided into two subscales: “fear of the short-term consequences of surgery” (5 items) and “fears of the long-term consequences of surgery” (3 items). The score for each item ranges from 0 to 10. To calculate the overall score, the sum of the scores for each item should be divided by the number of items in the instrument. Thus, higher values are associated with higher levels of fear^21^. Regarding the SFQ reliability, considering the total scores of both groups at the pre-intervention time, the result was α = 0.77, a value considered acceptable for this study^22^.

Regarding the physiological variables, HR and SBP/DBP were measured using the Omron® blood pressure and heart rate portable monitor (Japan). RR was identified by counting thoracic breathing movements for a period of 1 minute. Salivette^®^ (Sarstedt - Alemanha) swab in cotton was used to collect saliva for salivary cortisol identification. Samples were analyzed using the High Sensitivity Salivary Cortisol Ezyme Imunoassay Kit (1-3002; Salimetrics LLC, State College, PA), method ELISA/EIA. A system consisting of a disposable test strip and a portable analyzer, Cocoro Meter® (Nipro Corporation - Japan) was used to collect and analyze saliva for SAA identification.

The data obtained from the questionnaires were typed and organized in a spreadsheet using Microsoft Office Excel 2007. This spreadsheet was exported to the statistical program IBM - SPSS version 22, in which all the statistical analyzes of this study were conducted.

The variables for sociodemographic characterization of the sample were analyzed using descriptive statistics, with analyzes of distributions and frequencies. The distribution of the data was verified by the Shapiro-Wilk test. All variables presented non-normal distribution; therefore, non-parametric statistics were chosen to perform the other analyzes, considering p-values 0.05 as significant.

The non-parametric Mann-Whitney U test was used for the intragroup comparison of the physiological variables, state anxiety and surgical fears in the pre- and post-intervention moments. For the intergroup comparison, the non-parametric Wilcoxon test was used with repeated measures in the two moments.

The research was approved by the Research Ethics Committees of the Ribeirão Preto School of Nursing and of the Hospital das Clínicas of the Medical School of Ribeirão Preto (CAAE 11683313.9.0000.5393) and registered in the Clinical Trials platform (NCT02455128). After being informed about the study, participants were also informed about the anonymity and confidentiality of the data, and then signed the Informed Consent Form.

## Results

 The mean age of the participants was 58 years in the IG (SD = 11) and 57 years in the CG (SD = 15). Most of the participants had low level of education (incomplete and complete primary education), were married, Catholic and had a monthly family income between one and three minimum wages (Table 1). 

**Table 1:** Socio-demographic characteristics of patients in the preoperative period of colorectal surgery. Ribeirão Preto, SP, Brazil, 2015

Variables	n%	
	CG*	IG^†^
Gender		
Male	11 (44)	11 (44)
Female	14 (56)	14 (56)
Level of education		
≤ 4 years of education	9 (36)	15 (60)
5 - 11 years of education	14 (56)	9 (36)
> 11 years of education	2 (8)	1 (4)
Marital status		
Single	5 (20)	3 (12)
Married	13 (52)	17 (68)
Divorced/widowed	7 (28)	5 (20)
Monthly family income (minimum wages^‡^)		
1	2 (8)	6 (24)
2 - 3	17 (68)	17 (68)
4 - 5	4 (16)	1 (4)
6 - 10	2 (8)	0
No income	0	1 (4)
Religion		
Catholic	18 (72)	15 (60)
Protestant/ Spiritist and others	6 (24)	8 (32)
No religion, but spiritualized	1 (4)	2 (8)

* GC - Control Group; † IG - Intervention Group; ‡ Minimum wage was R$880,00 in the period of data collection.

The equivalence of the groups regarding the variables AAS, cortisol, HR, RR, SBP, DBP, state anxiety and surgical fears was verified in the pre-intervention moment. It is worth noting that the equivalence between the intervention and control groups regarding the variables of interest (AAS, cortisol, HR, RR, SBP, DBP, state anxiety and surgical fears) was verified in the pre-intervention moment was verified through the Mann-Whitney U test and there were no significant differences between groups. The means of the variables analyzed in the pre- and post-intervention are presented in Table 2.

There were no significant differences between the groups after the intervention, so at that moment the groups were equivalent in relation to the studied variables. Thus, the therapeutic listening intervention did not cause differences between the two groups under the conditions in which it was applied to the participants (Table 3).

**Table 2:** Means and Standard Deviations (SD) of the variables analyzed in the control group and intervention group in the pre- and post-intervention. Ribeirão Preto, SP, Brazil, 2015

Variables	CG* Mean (SD^‡^)		IG^†^ Mean (SD^‡^)	
	Pre	Post	Pre	Post
Salivary alpha-amylase (kU/L^§^)	137 (146.5)	123 (143)	174 (172.3)	163 (134)
Salivary cortisol (μg/dL^‖^)	0.39 (0.39)	0.29 (0.3)	0.33 (0.27)	0.30 (0.33)
Heart Rate (bpm^¶^)	80.32 (15)	76.44 (15)	77.48 (13)	77.44 (15)
Respiratory Rate (bpm**)	20.32 (5)	20.48 (4)	20.24 (5)	19.04 (5)
Systolic Blood Pressure (mmHg^††^)	124.5 (16)	122.2 (16)	119.5 (13)	122.6 (19)
Diastolic Blood Pressure (mmHg^††^)	77.2 (12)	76.3 (10)	75.4 (8)	77.8 (9)
State anxiety	37.4 (10.7)	36.2 (11.2)	37 (10.3)	36.3 (10.1)
Surgical fear	22.5 (18.9)	20.1 (19.6)	21.1 (19.8)	21.1 (19.7)

* CG - Control Group; † IG - Intervention Group; ‡ SD - Stardand deviation; § kU/L - kilounits per liter; ‖μg/dL - microgram per deciliter; ¶ bpm - beats per minute; ** bpm - breaths per minute; †† mmHg - millimeter of mercury

**Table 3:** Results of the Mann-Whitney test for comparison between the control and intervention groups at the post-intervention moment. Ribeirão Preto, SP, Brasil, 2015

Variables	Means		U^‡^	p^§^
	CG*	IG^†^		
Salivary Alpha-Amylase	19.57	26.28	177.5	0.086
Salivary Cortisol	14.13	12.64	73	0.966
Heart Rate	25.26	25.74	306.5	0.907
Respiratory Rate	28.32	22.68	242	0.157
Systolic Blood Pressure	25.68	25.32	308	0.930
Diastolic Blood Pressure	24.26	26.74	281.5	0.545
State Anxiety	24.86	26.14	296.5	0.756
Surgical Anxiety	24.96	26.04	299	0.793

* CG - Control Group; † IG - Intervention Group; ‡ U - Mann Whitney U; §p - Level of Significance

Two patients in the IG and three in the CG did not have enough saliva for the SAA analysis. The same occurred for cortisol with one participant in the IG and one in the CG. 

According to the results of Table 4, the changes between the pre- and post-intervention moments were not significant in the IG but were significant in the CG. In this group, before the intervention, the following means were obtained for salivary cortisol, HR and surgical fears: 0.39 μg/dL (SD=0.39), 80.32 bpm (SD=15) and 22.5 (SD=18.9), respectively. After the intervention, the means were 0.29 μg/dL (SD=0.3), 76.44 bpm (SD=15) and 20.1 (SD=19.6). Thus, there was a reduction in the values ​​of these variables from the pre-intervention moment to the post-intervention moment for the CG (Table 4).

**Table 4:** Results of the Wilcoxon tests for repeated measures (pre and post-intervention) for physiological variables, state anxiety and surgical fears. Ribeirão Preto, SP, Brazil, 2015

**Group**	**Variables**	NC*	**PC** ^**†**^	**V** ^**‡**^	**Means**		**Z** ^**§**^	**p** ^**‖**^
					**Negative Order**	**Positive Order**		
**CG** ^**¶**^	**Salivary Alpha-Amylase (n=22)**	**15**	**7**	**0**	**10.93**	**12.71**	**-1.218**	**0.223**
	**Salivary Cortisol (n=23)**	**17**	**6**	**0**	**12.03**	**11.92**	**-2.023**	**0.043**
	**Heart Rate (n=25)**	**15**	**8**	**2**	**13.83**	**8.56**	**-2.121**	**0.034**
	**Respiratory Rate (n=25)**	**5**	**9**	**11**	**9.30**	**6.50**	**-0.406**	**0.684**
	**Systolic Blood Pressure (n=25)**	**13**	**8**	**4**	**11.35**	**10.44**	**-1.114**	**0.265**
	**Diastolic Blood Pressure (n=25)**	**11**	**11**	**3**	**12.64**	**10.36**	**-0.406**	**0.684**
	**State Anxiety (n=25)**	**17**	**8**	**0**	**13.50**	**11.94**	**-1.817**	**0.069**
	**Surgical Fears (n=25)**	**15**	**5**	**5**	**10.87**	**9.40**	**-2.171**	**0.030**
**IG** ^**^	**Salivary Alpha-Amylase (n=23)**	**11**	**12**	**0**	**12.68**	**11.38**	**-0.046**	**0.964**
	**Salivary Cortisol (n=24)**	**8**	**3**	**0**	**5.88**	**6.33**	**-1.245**	**0.141**
	**Heart Rate (n=25)**	**10**	**12**	**3**	**11.75**	**11.29**	**-0.293**	**0.769**
	**Respiratory Rate (n=25)**	**9**	**5**	**11**	**8.22**	**6.20**	**-1.456**	**0.145**
	**Systolic Blood Pressure (n=25)**	**8**	**12**	**5**	**8.38**	**11.92**	**-1.423**	**0.155**
	**Diastolic Blood Pressure (n=25)**	**8**	**14**	**3**	**10.31**	**12.18**	**-1.435**	**0.151**
	**State Anxiety (n=25)**	**11**	**9**	**5**	**11.14**	**9.72**	**-0.656**	**0.512**
	**Surgical Fears (n=25)**	**10**	**10**	**5**	**11.60**	**9.40**	**-0.411**	**0.681**

*NC - Negative Classifications; †PC - Positive Classification; ‡V - Bonds; §Z - Statistics Z; ‖p - Level of Significance; ¶ CG - Control Group; ** IG - Intervention Group

Of the 50 participants in the study, 36 (72%) had their complete samples collected, in the morning and at night, with the volume of saliva necessary for the respective analyzes. For fourteen individuals, it was not possible to evaluate the circadian rhythm of salivary cortisol for different reasons: the amount of saliva collected was not enough in 3 patients (6%), the collection of the nocturnal period was not performed in 10 patients (20%) and one patient (2%) presented contamination of the sample. According to the specifications of the kit used for cortisol analyzes, the reference value for salivary cortisol at 11p.m. in normal subjects is between 0.007 and 0.115 μg/dL. Thus, of the 36 subjects evaluated, 15 (41.6%) had a circadian rhythm and 21 (58.3%) had no circadian rhythm.

## Discussion

In this study, we investigated the efficacy of therapeutic listening on state anxiety and surgical fears in preoperative colorectal cancer patients. Other non-pharmacological interventions have also been tested for their effectiveness in reducing anxiety in cancer patients and, in the circumstances in which they were performed, presented results that corroborate this study, since they also had no influence in reducing this feeling. A randomized controlled trial was conducted with the objective of testing the hypothesis that a multidisciplinary approach could improve understanding of the information provided by the anaesthesiologist and in turn, reduce anxiety in women undergoing surgery for breast cancer^23^. According to the results, there were no significant differences between the groups in the mean anxiety score before and after the intervention. However, for highly anxious patients (STAI ≥ 51), the STAI score significantly decrease in the multidisciplinary approach group when compared to the group that did not receive this intervention (p = 0.024).

It is worth noting that in the present study and in the aforementioned study^23^ the interventions were performed only once, different from other studies in which the interventions were performed over a longer period of time, such as for seven days^24^ or three weeks^25^, and which obtained positive results regarding the non-pharmacological interventions tested. Another factor to be taken into consideration is the dynamics of the patient care service in the place where the study was performed. Before participating in the study, the patients, who had a moderate anxiety score (Table 2), had already talked to the medical team about the treatment they would receive. According to the literature, the discussion with the medical team has been a coping strategy widely used by patients who are anxious about the surgical procedure they will undergo^26^. Therefore, it is possible that the clarifications previously offered by the medical team contributed to the levels of anxiety found among study participants.

On the topic of surgical fears, a study that aimed to identify the most common concerns about general anesthesia during the preoperative anesthetic clinic in different healthcare settings found that 88% of the patients experienced preoperative fear. The main causes were fear of postoperative pain (77.3%), fear of intraoperative awareness (73.7%) and fear of being sleepy postoperatively (69.5%)^27^. Excluding the patients who scored zero on the SFQ, the mean scores on the SFQ in both groups in the pre and post-intervention moments ranged from 20.1 to 22.5, respectively (Table 2). A possible explanation for the conclusion that therapeutic listening was not effective in reducing surgical fears can be attributed to the fact that the 30-minute time for performing the intervention, which was then followed by post-intervention data collection, was not enough for the patient to restore his emotional state after reflecting on his fears regarding the surgical procedure, 

According to the results of this study, in the pre-intervention moment the patients presented moderate levels of state anxiety and low levels of surgical fears (Table 2). It is possible that therapeutic listening would have a different effect in patients with higher scores, as occurred in a previously mentioned study, in which the intervention tested was effective for the reduction of anxiety only in patients who had high levels of anxiety^23^.

There are reports that the communication between researcher and patient during the data collection moments can contribute to decrease the anxiety levels of the patients, even in individuals of the control groups^28^. Therefore, the relationship established between the researcher and the patients in these moments may have contributed to the results obtained in the present study, which included a decrease in salivary cortisol, HR and surgical fears in the CG (Table 4). In the nursing care process, actions must go beyond the technical act and be based on the permanent relationship with each other, including touch, communication, physical care and respect, which are fundamental aspects for the promotion of patient well-being^29^.

A possible justification for the changes found in the CG not being observed in the IG is that, in the latter, the issues discussed raised reflections about the experience of hospitalization for the treatment of the disease, which may have made the patients remember their concerns about such a situation. In order to assist the patient in the management of feelings such as anxiety and fear, there may be a need for more time for therapeutic listening, with a greater number of meetings between nurse and patient. In adittion, the 30-minute time for the evaluation of these parameters may have been small for processing the emotions raised in the nurse-patient interaction, affecting the parameters observed in the second evaluation.

Regarding the circadian rhythm of cortisol, it should be considered that tumor patients may exhibit nearly normal or markedly altered circadian rhythms^30^. Variations in the circadian rhythm of cortisol in patients with colorectal cancer have already been reported in the literature^31^ ^-^ ^32^. These variations in the circadian rhythm of cortisol in cancer patients may be due to physical factors such as fatigue^33^ or dysregulation of the hypothalamic-pituitary-adrenal axis^34^. In addition, cortisol secretion patterns change in both acute and chronic ilness, as a result, for example, of the action of inflammatory mediators^35^. Thus, changes in the circadian rhythm of cortisol in cancer patients may be due to the disease itself, which may be responsible for modifications in the components of the circadian system^36^.

## Conclusion

The objective of this study was to evaluate the effect of therapeutic listening on state anxiety and surgical fears preoperative colorectal cancer patients. In the conditions under which the intervention was conducted and considering the state anxiety and surgical fears found in the pre-intervention moment, it was not possible to observe a reduction in the levels of the physiological and psychological variables related to the therapeutic listening.

However, the meeting between the researcher and the patients of the CG for data collection, when there was only the contact without stimulus to reflect on the situation they were experiencing (intervention), may have allowed the reduction of salivary cortisol, HR and surgerical fears. Thus, on the day before the surgical procedure, the care and attitudes offered by the nurse to the patient in this study were efficient in reducing the variables assessed.

One factor that must be taken into account was the time of interaction with the researcher for the therapeutic listening, which was the period between the two moments of data collection (pre-intervention and post-intervention), when the patients were invited to reflect on their situation and to expose their feelings and thoughts. The 30-minute time may have been insufficient for the patient to perform this task and then return to a regular emotional state. This may have interfered in the results of the studied variables and, consequently, in their measurements. Possibly, a greater number of sessions could produce different results. However, in the preoperative context in which this study was performed this would not be possible, since the patients were admitted as close as possible to the time of surgery. In another context, such as in an outpatient service, it would have been possible to have more encounters with the patients.

In addition, it is possible that a period longer than 30 minutes between the intervention and the collection of the post-intervention data can provide smaller scores of the studied variables, since, with a longer time after the therapeutic listening, the patients would have time to process their emotions and, thus, could possibly present emotional conditions closer to the desirable. These aspects deserve further study.

The present study highlights the use of therapeutic listening as a nursing intervention in patients with colorectal cancer in the preoperative period, considering that the use of this intervention may enable a patient-centered information collection, since therapeutic listening puts the patient, and not the disease, as the center of the actions.
